# White paper – A proposal towards objective biomechanical metrics as novel endpoints to document improvements in musculoskeletal function and mobility

**DOI:** 10.1016/j.ocarto.2025.100648

**Published:** 2025-07-05

**Authors:** Sónia A. Alves, Nicholas M. Brisson, Alison N. Agres, Mark Heyland, Ali Mobasheri, David J. Hunter, Tobias Winkler, Georg N. Duda

**Affiliations:** a Julius Wolff Institute, Berlin Institute of Health at Charité – Universitätsmedizin Berlin, Berlin, Germany; b Research Unit of Health Sciences and Technology, Faculty of Medicine, University of Oulu, Oulu, Finland; c Sydney Musculoskeletal Health, Arabanoo Precinct, Kolling Institute, Faculty of Medicine and Health, The University of Sydney, Sydney, Australia; d Berlin Institute of Health Center for Regenerative Therapies, Berlin Institute of Health at Charité – Universitätsmedizin Berlin, Berlin, Germany; e Center for Musculoskeletal Surgery, Charité – Universitätsmedizin Berlin, Berlin, Germany

## Abstract

**Figure:**
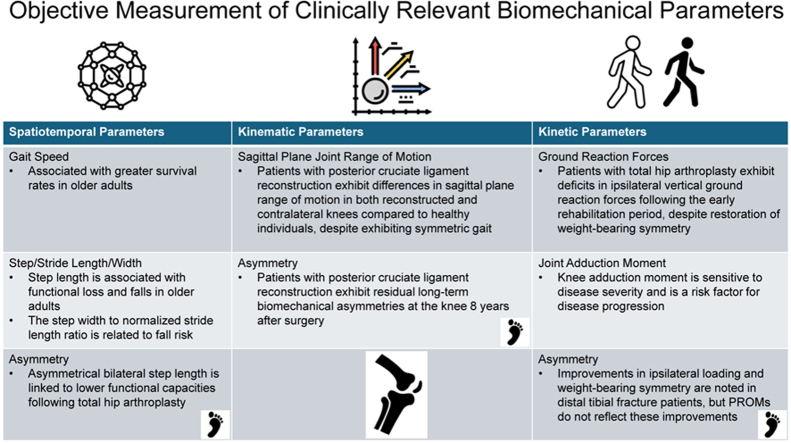

## 1 Introduction

Musculoskeletal (MSK) conditions are the leading cause of rehabilitation needs, contributing to direct healthcare costs, work disability, and reduced well-being. The Global Burden of Disease 2019 reported a high prevalence of MSK conditions, affecting an estimated 1.71 billion people worldwide, with projections suggesting a 115 ​% increase from 2020 to 2050 [1].

MSK conditions also elevate the risk of chronic diseases. For instance, osteoarthritis (OA), the most common MSK condition amongst older adults, is associated with a higher likelihood of developing cardiovascular disease [2]. The relationship between MSK conditions and systemic diseases complicates the assessment of mobility, which is integral to overall well-being. This interplay is particularly important in clinical trials, where the primary goal is to develop effective therapies for MSK disorders.

Functional impairments in MSK conditions are often unilateral, with treatment strategies aiming to restore pre-condition function. However, existing functional assessments are typically task-dependent and lack specificity in evaluating the affected limb. For instance, physical performance-based tests (PBTs) commonly measure the time to complete a task (e.g., Timed Up and Go Test), repetitions performed within a set time (e.g., sit-to-stand test), or the distance travelled in a set time (e.g., Six Minute Walk Test), as indicators of overall MSK function. While these, along with patient-reported outcome measures (PROMs), are valuable for evaluating intervention efficacy, they often fail to capture underlying movement mechanics, which are critical for evaluating true functional recovery of deterioration. Patients would substantially benefit from complimentary, objective, and quantitative biomechanical data. This is particularly important for populations where self-reports may be unreliable, such as elderly individuals with cognitive decline and children with communication barriers. In the elderly, gait and balance assessments can offer valuable insights into mobility decline, while in pediatric populations, motion analyses during play-based activities provide a more ecologically valid assessment of MSK function.

For decades, movement-based biomechanical metrics – objective measures of body movement, forces and mechanics – have provided valuable insights into MSK rehabilitation. This is particularly relevant for OA, where traumatic joint injuries, such as articular fractures or cruciate ligament tears, greatly increase the risk of post-traumatic OA. By identifying abnormal movement patterns, biomechanical metrics can help predict OA onset and support early interventions aimed at preventing degenerative changes. When properly implemented, these metrics provide greater validity and reliability than PROMs for assessing MSK function during rehabilitation in clinical trials.

### 1.1 Problem statement: limited adoption of biomechanical metrics in clinical trials

Although biomechanical metrics are valuable indicators of recovery, their use in clinical trials remains limited, resulting in an incomplete understanding of MSK function and treatment efficacy. While PROMs and PBTs (e.g., Timed Up and Go, sit-to-stand) offer important information, they often lack the sensitivity to detect subtle yet clinically relevant functional changes, particularly during the early stages of recovery [3]. PROMs and PBTs typically struggle to differentiate the impact of conditions such as lower back pain, diabetic neuropathy, and foot disorders. In contrast, biomechanical measures, with their joint-specific focus, provide a distinct advantage in assessing how comorbidities affect mobility.

## 2 Proposed framework for adopting biomechanical metrics in MSK clinical trials

Integrating biomechanical measures into clinical trials deepens the understanding of therapeutic effects, optimizes patient selection, and supports evidence-based guidelines. However, several challenges hinder their widespread adoption, including high equipment costs, complex data collection procedures, and time constraints. A lack of standardized protocols and validated metrics further complicates their implementation, alongside clinical and regulatory barriers such as limited normative data and uncertainties around clinical significance. To overcome these obstacles, we propose a framework that facilitates the adoption of objective biomechanical measures as key clinical endpoints, especially in trials targeting improved MSK function and mobility.

Table 1 provides a structured overview of key biomechanical parameters relevant to different MSK conditions. These metrics fall into three main categories: (i) spatiotemporal (measures related to space and time, like gait speed and step length); (ii) kinematic (movement-based parameter such as joint angles, velocities, and accelerations); and (iii) kinetic (force-related metrics like ground reaction forces and joint loading). Several factors may influence biomechanical outcomes in MSK trials, which are outlined below.

**Table 1:** Description of biomechanical parameters (including clinically relevant examples) that are objectively measured during activities of daily living, easily interpretable, and applicable as clinical endpoints. The biomechanical data include spatiotemporal, kinematic, and kinetic parameters.

**Spatiotemporal parameters**
Gait speed • Gait speed is associated with greater survival rates in older adults [10].
Step/stride length/width • Step length is associated with functional loss and falls in older adults [14]. • The step width to normalized stride length ratio is related in older adults [14].
Asymmetry • Asymmetrical bilateral step length is linked to lower functional capacities following total hip arthroplasty [15].
**Kinematic parameters**

Sagittal plane joint range of motion • Patients with posterior cruciate ligament reconstruction exhibit differences in sagittal plane range of motion in both their reconstructed and contralateral knees compared to healthy individuals, despite exhibiting symmetric gait [4].
Asymmetry • Patients with posterior cruciate ligament reconstruction exhibit residual long-term biomechanical asymmetries at the knee 8 years after surgery [8].
**Kinetic parameters**

Ground reaction forces • Patients with total hip arthroplasty exhibit deficits in ipsilateral vertical ground reaction forces following the early rehabilitation period, even though weight-bearing symmetry is restored [3].
Joint moments • In persons with knee OA, the knee adduction moment is sensitive to disease severity and is a risk factor for disease progression [13].
Asymmetry • Critical improvements in ipsilateral loading and weight-bearing symmetry are noted in distal tibial fracture patients, although PROMs do not reflect these improvements [12].

### 2.1 Baseline assessments for outcome interpretation

Pre-interventional measurements, when available, help establish baseline joint function and mobility, serving as a reference for evaluating recovery and treatment response. When baseline data are unavailable (e.g., trauma cases), alternative approaches such as accounting for key patient characteristics (e.g., age, sex, body mass, physical activity level, overall health status), leveraging historical data, or acquiring early post-injury measurements may help to improve interpretability.

### 2.2 Frequent early follow-up measurements

Recovery of weight-bearing symmetry and pre-operative activity levels often occurs within weeks post-intervention (e.g., 6–12 weeks after primary unilateral total hip arthroplasty) [3]. Early and frequent follow-up assessments are crucial for capturing these rapid functional changes and monitoring recovery trajectories. This approach enhances the detection of functional improvements or declines, which may be missed by traditional designs with sparse follow-ups, especially in both acute (e.g., bone fractures) and chronic (e.g., joint arthroplasty) conditions.

### 2.3 Comparison to healthy peers rather than the contralateral limb

Individuals with unilaterally mobility impairments (e.g., knee ligament injuries, bone fracture) often exhibit prolonged limb asymmetries lasting weeks to years after trauma. While some asymmetries resolve naturally over time, apparent symmetry may result from compensatory maladaptation of the unaffected limb rather than true recovery, resulting in movement patterns different from those of healthy, asymptomatic controls [4]. For example, after Achilles tendon rupture, ankle range of motion during walking may appear restored; however, this often reflects excessive dorsiflexion compensating for limited plantarflexion [5]. Similarly, patients with posterior cruciate ligament reconstruction may show no biomechanical differences between their reconstructed and contralateral knees during self-paced walking, but significant deviations compared to healthy controls [4]. Additionally, pre-existing conditions can confound baseline comparisons. If a condition has already altered movement patterns, pre-intervention values may not serve as valid reference points for evaluating recovery. Since even healthy individuals exhibit minor asymmetries during daily activities [3,6], recovery assessments should compare results to healthy peer data rather than assuming perfect symmetry or relying solely on contralateral limb comparisons. This approach provides a more meaningful benchmark for evaluating functional restoration.

### 2.4 Activity assessment beyond gait analysis

A comprehensive assessment of functional deficits requires analyzing activities beyond basic gait analysis [3,7,8]. Many MSK conditions affect diverse movement patterns, which may be better revealed through biomechanically more challenging tasks. Activity selection should align with the clinical question and consider the site, severity, and symptoms of the condition. For instance, sit-to-stand transitions can effectively detect weight-bearing asymmetries in patients recovering from primary unilateral total hip arthroplasty [3]. Evaluating unilateral loading during such tasks ensures that observed symmetry is not falsely attributed to poor bilateral loading patterns.

## 3 Conclusions

As patient-centered definitions of success become more prominent, further efforts are needed to optimize outcomes. Expanding the use of biomechanical measures can help detect subtle yet clinically meaningful changes in impairments that may not be fully captured by PROMs or PBTs alone.

To establish biomechanical metrics as clinical endpoints, rigorous validation is essential despite inherent challenges. Validation includes correlating biomechanical metrics with established clinical outcomes (e.g., knee moments linked to cartilage loss); aligning biomechanical metrics with PROMs; demonstrating predictive validity (e.g., asymmetry predicting reinjury risk), and confirming responsiveness to interventions. Defining clinically meaningful change is also critical. This involves using Minimal Detectable Change to account for measurement error [9]; Minimal Clinically Important Difference to capture functional improvements (e.g., a 0.1 m/s gait speed increase is associated with improved survival [10]); and applying clinical thresholds to identify biomechanical risk factors (e.g., knee loading predicting OA progression [11]). Furthermore, sensitivity and specificity to particular diseases, like OA, must be demonstrated. For example, while systemic diseases may not present with movement asymmetries, unilaterally manifested MSK conditions such as OA can significantly affect biomechanical symmetry.

To promote wider adoption of biomechanical metrics, it is essential to standardize protocols and establish normative versus pathological reference values. When employing novel technologies (e.g., wearables) for biomechanical data collection, rigorous validation is critical before clinical or trial use. Once validated, these tools can bridge experimental findings with regulatory requirements, maintaining ecological validity and enhancing the generalizability of results to real-world settings.

We recommend prioritizing objective biomechanical metrics in gait or other activities of daily living as key endpoints for assessing physical function and mobility in MSK conditions. These should complement PROMs and PBTs, which remain widely used in clinical trials. Biomechanical metrics should be validated against both generic and condition-specific measures of function and mobility to assess their responsiveness to change and diagnostic ability. Clinical research has already shown that biomechanical metrics can effectively distinguish between healthy and diseased states [3,4,8,12,13]. The proposed framework provides guidance on their implementation in clinical trials, facilitating their integration into evidence-based research and practice.

## Declaration of author contributions

All authors made substantial contributions to all of the following: (1) the conception and design of the study, or acquisition of data, or analysis and interpretation of data, manuscript, (2) drafting the article or revising it critically for important intellectual content, and (3) final approval of the version to be submitted. SAA, NMB, ANA, and MH drafted the initial manuscript. SAA, NMB and GND take responsibility for the integrity of the work as a whole, from inception to finished article.

## Declaration of funding

This work was supported in part by research funding from the German Research Foundation (DFG – Deutsche Forschungsgemeinschaft, BR 6698/1-1 (NMB); AG 293/2-1 (ANA); Project ID 427826188 - SFB 1444/CRC 1444 (MH, GND), FOR 5177 (GND, SA)) and in part by the ERC Adv Grant “Immuno-Mechanics” (GND). Consortium funding was received from the European Union Horizon Europe Programme under grant agreement number 101095635 (PROTO – Advanced PeRsOnalized Therapies for Osteoarthritis – Tackling inflammation to improve patient outcomes (NMB, AM, TW, GND)) and grant agreement number 101137315 (ENCANTO – ENgineered CArtilage from Nose for the Treatment of Osteoarthritis (AM)). Views and opinions expressed are however those of the author(s) only and do not necessarily reflect those of the European Union or the European Health and Digital Executive Agency. The European Union or granting authority cannot be held responsible for them. AM also acknowledges financial support from the European Structural and Social Funds through the Research Council of Lithuania (Lietuvos Mokslo Taryba), according to the Program Attracting Foreign Researchers for Research Implementation (grant number 01.2.2-LMT-K-718-02-0022) plus networking grant support from the European Cooperation in Science and Technology (COST) Association, Action CA21110 – Building an open European Network on OsteoArthritis research (NetwOArk). DJH is employed by the University of Sydney and Royal North Shore Hospital. His salary support for the University of Sydney is supported by Arthritis Australia and an NHMRC Investigator Grant Leadership 2 (#1194737).

## Declaration of competing interest

ANA and MH have no conflicts of interest to declare. SAA is currently employed by Life Molecular Imaging GmbH. NMB is Associate Editor for *Osteoarthritis and Cartilage Open*. AM served as former President of the Osteoarthritis Research Society International (May 2019–May 2022) and a member of the Osteoarthritis Research Society International Board of Directors from 2013 to 2022. AM currently serves as an advisor for the World Health Organization Collaborating Center for Public Health Aspects of Musculoskeletal Health and Aging, Member-at-Large on the Steering Committee Osteoarthritis Action Alliance, and Member of the Scientific Advisory Board of the European Society for Clinical and Economic Aspects of Osteoporosis, Osteoarthritis and Musculoskeletal Diseases (ESCEO). AM provides scientific consulting on the advisory boards for HALEON (Global Pain Faculty, Naturals Advisory Board), Sanofi, Sanofi Consumer Healthcare (Opella Healthcare), Kolon TissueGene, Enlivex, Pacira BioSciences, Contura, Chondrometrics, Aptissen SA, Synartro AB, Contura AB, ICM (South Korea), Kangstem, Peptinov, Pluri, Chondropeptix and the California Institute for Regenerative Medicine, California's Stem Cell Agency. DJH is the Editor of the osteoarthritis section for UpToDate and co-Editor-in-Chief of *Osteoarthritis and Cartilage*. DJH provides consulting advice on scientific advisory boards for Haleon, TLCBio, Novartis, Tissuegene, Sanofi, Enlivex. TW is co-founder and co-chair of the Advanced Therapies in Orthopaedics Foundation – ATiO. TW provides scientific consulting on the advisory boards of Relive, Enlivex, Liposphere, Ossium, Heraeus Medical and Pluri. GND provides scientific consulting on advisory boards of Pluri Inc., Heraeus Medical, BBraun Aesculap, Stryker, J&J DePuy, Implantcast and B&C.
